# Green Synthesis and Characterization of Silver and Gold Nanoparticles Using *Echinophora platyloba* Extract and Evaluation of Their Anti-Inflammatory and Antioxidant Properties

**DOI:** 10.1155/omcl/4421985

**Published:** 2025-03-06

**Authors:** Maryam Azadmanesh, Mohammad Foad Noorbakhsh, Saeed Nazifi, Milad Faraji

**Affiliations:** ^1^Department of Basic Sciences, School of Veterinary Medicine, Shiraz University, Shiraz, Iran; ^2^Department of Clinical Sciences, School of Veterinary Medicine, Shiraz University, Shiraz, Iran

**Keywords:** anti-inflammation, antioxidant, *Echinophora platyloba*, gold nanoparticle, green synthesis, silver nanoparticle

## Abstract

This study intends to investigate the green synthesis of silver (Ag) and gold (Au) nanoparticles (NPs) using *Echinophora platyloba* extract and to evaluate the antioxidant and anti-inflammatory effects of the synthesized NPs and the extract. In this study, aqueous and hydroalcoholic extracts of *E. platyloba* were prepared, which were used for the biosynthesis of Ag and Au NPs. Dynamic light scattering (DLS), zeta potential analysis, transmission electron microscopy (TEM), Fourier transform infrared (FT-IR) spectroscopy, UV-Vis spectroscopy, and X-ray diffraction (XRD) methods were used to characterize the green NPs. The antioxidant effect of the NPs was estimated using in vitro methods, including reducing power (RP), ferric reducing/antioxidant power (FRAP), and 2,2-diphenyl-1-picrylhydrazyl (DPPH). To evaluate the anti-inflammatory and antioxidant activity of *E. platyloba* extract and Ag and Au NPs, we used the carrageenan method. In our experiment, the extract and the synthesized NPs were administered orally to the mice 2 h before the carrageenan injection. The subsequent inhibition of inflammation and reduction of paw thickness were quantified. To evaluate their antioxidant effect, malondialdehyde (MDA), and total antioxidant capacity (TAC) levels were measured. Levels of pro-inflammatory cytokines, interleukin-6 (IL-6) and tumor necrosis factor-*α* (TNF-*α*), were also quantified. In this study, the results indicate that the synthesized Ag and Au NPs have antioxidant and anti-inflammatory effects. The most promising results were observed in the groups that received the Ag NPs.

## 1. Introduction

Nanotechnology fuses different fields of science, like engineering, information technology, material science, and life science. Several researches have also shown promising results of nanotechnology for human health, especially for treating cancer [1]. Nanoparticles (NPs) are very small structures that usually measure between 1 and 100 nm [2]. NPs possess unique chemical and physical characteristics in comparison with their bulk form. This difference is primarily because of their tiny size and high surface-to-volume ratio. Additionally, interparticle distance, the features of the protecting organic shell, and the shape of the NPs are other factors affecting the properties of the NPs [3, 4].

NPs have been created using different methods, including physical, chemical, and biological processes. However, most of the mentioned methods are expensive and hazardous to public health because of the involvement of toxic substances such as solvents, reducers, and precursors [5]. Green synthesis is an area of study that concentrates on the creation of NPs using plants, and this field is quickly advancing because of the increasing success and facility of the generation of NPs in this method. The biogenesis of metal NPs is an eco-friendly method that does not need to use toxic and expensive chemicals [4, 6]. Different extracts derived from various parts of plants, such as leaves, bark, seeds, peels, petals, rhizomes, gums, roots, and fruit, are being used to reduce metallic precursors to NPs [7]. The capping and reduction of green NPs are done by phytochemicals, like phenolics, saponins, terpenoids, flavonoids, and the functional groups in these compounds [8]. These NPs have remarkable biomedical properties such as targeted drug delivery, antimicrobial activity, wound healing, and bioimaging. Additionally, they have shown acceptable efficacy in fighting cancer, inflammation, and microbial growth [9].

Silver (Ag) and gold (Au) NPs possess distinctive biological characteristics. Because of the high biocompatibility of Au NPs, they have gained recognition in biomedical applications. Due to their characteristics, they are being used for diagnostic and therapeutic purposes [10, 11]. Ag NPs have anti-HIV [12], antiplatelet [13], antifungal, antiviral, anti-inflammatory, antiangiogenic, anticancer, and antioxidative effects [14].

*Echinophora platyloba* (*E. platyloba*) belongs to the Apiaceae family and is known as Khusharizeh, Khusharozeh, or Tagh Touraq in Persian. This plant has a pleasant aroma and taste. Secondary metabolites of the *Echinophora* genus include alkaloids, saponins, and flavonoids [15]. The aerial parts of *E. platyloba* add flavor to cheese and yogurt. This plant is also known for its ability to alleviate flatulence and freshen the air [16]. *Echinophora platyloba* contains phenolic, flavonoid, and terpenoid compounds. Various studies have identified the flavonoids myricetin, myrcene, and quercetin. Previous research has also confirmed the presence of the terpenoids ocimene, phellandrene, limonene, pinene, and carene [17]. Furthermore, these phenolic, flavonoid, and terpenoid compounds play an important role in the synthesis and stabilization of NPs, and there is potential for antioxidant and anti-inflammatory effects due to the capping properties of flavonoids [18]. *Echinophora platyloba* extract has hepatoprotective [19], antioxidant, antidepressant [20], antibacterial [21], anticancer [22], and antifungal effects [23].

The purpose of this essay is to explore the green synthesis of Ag and Au NPs using *E. platyloba* extracts. The green NPs will then be evaluated for their antioxidant and anti-inflammatory properties.

## 2. Materials and Methods

### 2.1. Preparation of Plant Extracts

*Echinophora platyloba* was collected from Khorasan Province in the northwest of Iran and approved. The plant parts were washed twice with distilled water and then shade-dried for 10 days. The dried material was then ground with an electric blender–grinder (Philips HR2041, Netherlands). To prepare the boiled extract, 50 g of this powder were heated in 400 mL of distilled water for 10 min using a hot plate. The extract was then filtered through Whatman No. 1 filter paper. For preparing the aqueous extract, 100 g of the plant powder were soaked in 500 mL of distilled water for 24 h at room temperature. The extract was then filtered through Whatman No. 1 filter paper. To prepare the hydroalcoholic extract, 100 g of powdered plant were mixed with 250 mL of 50% methanol (Sigma–Aldrich, USA) in a 500 mL Erlenmeyer flask and shaken at 120 rpm for 24 h. After that, the solution was filtered, and the solvent was evaporated to obtain a crude extract. We turned it into a powder using a freeze dryer. The resulting powder was stored in an airtight container at 4°C.

### 2.2. Synthesis of Green Au and Ag NPs

To create the Au and Ag solutions, HAuCl_4_·3H_2_O (Merck, Germany) and AgNO_3_ (Merck, Germany) crystals were separately dissolved in distilled water to make 1 and 0.5 mM stock solutions, respectively. The *E. platyloba* stock solution was made by dissolving 20 mg of *E. platyloba* in 10 mL of distilled water. To prepare Au and Ag NPs, 9.5 mL of each stock solution was mixed with 0.5 mL of the *E. platyloba* solution in a small glass bottle. Within 2 h, the yellowish Au solution became purple, and the colorless Ag solution became dark yellow.

### 2.3. Determination of Total Phenolics and Flavonoids

The total phenolics were measured by spectrophotometric analysis using Folin–Ciocalteu's phenol reagent (Merck, Germany) [24, 25]. Initially, 200 mL of diluted sample or gallic acid (Sigma–Aldrich, USA) standard was mixed with 2.6 mL of distilled deionized water. Then, at time zero, 200 mL of Folin–Ciocalteu's phenol reagent was added and thoroughly mixed. Two milliliter of 7% (*w*/*v*) Na_2_CO_3_ (Merck, Germany) solution was added after 6 min and mixed. The mixture was then incubated at room temperature for 90 min, and the absorbance at 750 nm against a prepared blank was measured using a spectrophotometer (UNICO 2100, USA). To make the blank, 200 mL of 50% (*v*/*v*) methanol (Sigma–Aldrich, USA) was used instead of the sample. A standard solution of gallic acid in 50% (*v*/*v*) methanol (Sigma–Aldrich, USA), with concentrations of 10, 30, 60, and 100 mg/L, was utilized to create a standard curve. The content of total phenolics was expressed as µg of gallic acid equivalent (GAE) per mg of dry sample. All samples were analyzed in triplicate.

The total flavonoids were evaluated via the aluminum chloride method [26, 27]. One milliliter of diluted sample or standard solutions of quercetin (Sigma–Aldrich, USA; 20–400 mg/L) and 4 mL of ddH_2_O were added to a 10 mL volumetric flask. At the beginning, 0.3 mL of 5% NaNO_2_ (Merck, Germany) was added to the flask. After 5 min, 0.3 mL of 10% AlCl_3_ (Sigma–Aldrich, USA) was added, followed by the addition of 2 mL of 1 M NaOH (Merck, Germany) at 6 min. The reaction flask was then immediately diluted to volume by adding 2.4 mL of ddH_2_O and thoroughly mixed. The resulting pink-colored mixture was measured for absorbance at 510 nm compared to the prepared water blank. The total flavonoid content (TFC) of the extract was expressed as µg/mg quercetin equivalents (QEs) on a dry weight (DW) basis. The samples were analyzed in triplicate.

### 2.4. Characteristics of Synthesized NPs

UV-Vis spectroscopy was used to record the absorption spectra of the synthesized Ag and Au NPs. The measurements were taken at the 1^st^, 2^nd^, and 24^th^ h after NP synthesis, in the range of 200–800 nm, and a blank reference of distilled water was used. Our measurements were performed using a UV-Vis spectrophotometer (Spekol 100, Germany).

X-ray diffraction (XRD) verified the crystallinity of the synthesized NPs; this technique was performed using a diffractometer (D8 ADVANCE, Bruker, Germany) to obtain a diffractogram from the dried thickness of the sample on a microscope slide.

Transmission electron microscopy (TEM) analysis was done utilizing a transmission electron microscope (Philips CM-10, Netherlands). The samples were prepared by placing a drop of the primary sample on a holey carbon-coated copper grid. This grid was dried at 80 °C for 6 h in an oven.

Dynamic light scattering (DLS) and zeta potential analysis were employed to study the hydrodynamics of NPs. The DLS technique provides information about the median size and distribution of sizes of NPs present in a liquid. NPs are suspended in an aqueous solution at room temperature to measure the zeta potential. These techniques were performed using a NP analyzer (SZ-100, Horiba, Japan).

Fourier transform infrared (FT-IR) measurements were conducted to discover the functional groups present in the synthesized NPs, which are responsible for the reduction and stabilization of the NPs. In this study, a FT-IR spectrometer (Tensor II, Bruker, Germany) was used.

### 2.5. In Vitro Antioxidant Effects

To evaluate the reducing power (RP) of extracts, an adjusted version of the method reported by Oyaizu [28] was performed. One milliliter of methanolic extract from *E. platyloba*, as well as Au and Ag NPs at varying concentrations, was combined with 2.5 mL of phosphate buffer solution (0.2 mM, pH 6.6) and 2.5 mL of 1% potassium ferrocyanide (Merck, Germany). After being incubated for 20 min at 50°C in a water bath, 2.5 mL of 10% trichloroacetic acid (Merck, Germany) was added, and the mixture was centrifuged at 3000 rpm for 10 min. The supernatant was combined with 2.5 mL of distilled water and 0.5 mL of 0.1% FeCl_3_ (Merck, Germany), and its absorbance was measured at 700 nm using a spectrophotometer. The blank was prepared by substituting the same quantity of diluted extract with methanol. The findings were expressed as milligram equivalents of quercetin per milligram of DW. Triplicate measurements were taken for each sample.

To determine the 2,2-diphenyl-1-picrylhydrazyl (DPPH) of samples, free radical scavenging activity, a procedure was adopted from the method developed by Hatano et al. [29], with some modifications. In this experiment, 2 mL of a methanolic solution of DPPH (Sigma–Aldrich, USA; 0.1 mM) was combined with 0.2 mL of samples prepared with methanol to reach a volume of 3 mL. After 60 min, the absorbance was measured at 517 nm. Methanol was utilized as a blank and ascorbic acid (Merck, Germany) was used as the standard. Triplicate measurements were taken for each sample.

  $$\begin{array}{c} \text{DPPH scavenging effect}\left(\%\right)=\left[\left(A_{0}-A_{\text{t}}\right)/A_{0}\right]\times 100. \end{array}$$

*A*_0_ is the absorbance of the control and *A*_t_ is the absorbance of the sample.

The ferric reducing/antioxidant power (FRAP) assay was conducted based on the method of Benzie and Strain [30]. First, the FRAP reagent was prepared by mixing 10 parts of a 300 mM sodium acetate buffer solution at pH 3.6, one part of 10 mM TPZT (Merck, Germany), and one part of 20 mM FeCl_3_ (Merck, Germany). Then, 0.2 mL of each sample (*E. platyloba*, Au NPs, and Ag NPs) was mixed with 3.8 mL of the FRAP reagent. After mixing, the resulting solution was incubated at 37°C for 30 min. The increase in absorbance was measured at 593 nm using a spectrophotometer. To prepare the blank, the same quantity of samples was replaced with methanol. The outcomes were reported as milligram equivalents of FeSO_4_ (Merck, Germany) per milligram. Triplicate measurements were taken for each sample.

### 2.6. In Vivo Antioxidant and Anti-Inflammatory Effects

The anti-inflammatory and antioxidant activities of *E. platyloba* extract, Ag NPs, and Au NPs were assessed based on the method presented by Winter, Risley, and Nuss [31] with some adjustments. Forty five adult male BALB/c mice, with an average weight of 35 g and an age of 8–10 weeks, were obtained from Shiraz University of Medical Sciences. Before the experiment, they were monitored for a week to confirm their health status and acclimatize to the environment. The animal cages were maintained under standard conditions, including a temperature of 22 ± 3°C, 60% ± 5% humidity, and a 12-h light/dark cycle. Commercial standard pellets were used for feeding, and the mice had free access to water. Mice were split into eight groups (*n* = 5): *E. platyloba* extract (15 and 30 mg/kg), green Ag NPs (0.3 and 0.6 mg/kg), green Au NPs (0.3 and 0.6 mg/kg), commercial Ag NPs (US Research, USA), indomethacin (the reference anti-inflammatory drug; 10 mg/kg), and 0.9% saline, which were administered orally 2 h before carrageenan (Sigma–Aldrich, USA) was injected into the sub-plantar area of the right hind paw of the mice. Dose selection for the extract and NPs was based on the results of the pilot study. Before and at the 1^st^, 2^nd^, 3^rd^, and 4^th^ h after injection, the thickness of the right hind paws of the mice was measured utilizing a Vernier caliper. At the 4^th^ h, the mice were anesthetized using ketamine (100 mg/kg, i.p., Alfasan, Netherlands) and xylazine (10 mg/kg, i.p., Alfasan, Netherlands), and then euthanized using carbon dioxide (CO_2_) inhalation. Both hind paws were harvested from the tarsocrural joint. We weighed the tissue and then froze it with liquid nitrogen and crushed it with a mortar. Next, we combined the crushed tissue with PBS buffer (1 M, pH = 7.4) at a ratio of 1:10. Then, we centrifuged it for 10 min at 6000 rpm at 6°C. Finally, we collected the supernatant. The supernatant was utilized to assess antioxidant (total antioxidant activity and malondialdehyde (MDA)) and anti-inflammatory activities (tumor necrosis factor-*α* (TNF-*α*) and interleukin-6 (IL-6)).

MDA, as a marker of lipid peroxidation in the tissue, was measured using a commercial kit (ZellBio GmbH, Germany); the microplate was read by a microplate reader (MQX200R2, Biotek Instruments, Burlington, VT, USA), as instructed.

Total antioxidant capacity (TAC) was measured using a commercial kit (ZellBio GmbH, Germany), and the microplate was read by a microplate reader (MQX200R2, Biotek Instruments, Burlington, VT, USA), as instructed.

The activity of TNF-*α* was assessed utilizing a commercial enzyme-linked immunosorbent assay kit (Abcam Company, USA), and the microplate was read by a microplate reader (MQX200R2, Biotek Instruments, Burlington, VT, USA), as instructed.

The IL-6 level was evaluated utilizing an enzyme-linked immunosorbent assay kit (Abcam Company, USA), and then the microplate was read by a microplate reader (MQX200R2, Biotek Instruments, Burlington, VT, USA), as instructed.

### 2.7. Statistical Analysis

The data obtained were analyzed using GraphPad Prism software v10.0 (USA) and reported as mean ± standard error of the mean (SEM). Statistical comparisons were conducted using appropriate methods, including one-way ANOVA followed by Tukey's post hoc test. A significance level of *p* < 0.05 was considered statistically significant in all comparisons.

## 3. Results

### 3.1. Phenolic Content

The total phenol content (TPC) of the *E. platyloba* extract was 25.84 ± 7.439 µg GAE/mg dry extract. The phenol content of green Ag NPs and green Au NPs was 20.44 ± 1.404 and 18.42 ± 1.265 µg GAE/mg dry extract, respectively. The TPC of commercial NP Ag was 0.3340 ± 0.5768 µg GAE/mg dry extract (Table 1).

### 3.2. Flavonoid Content

The TFC of the *E. platyloba* extract was 51.64 ± 10.15 µg QE/mg dry extract. This content for the green Ag NPs and green Au NPs was significantly lower (*p* < 0.05) at 26.99 ± 0.5015 and 25.80 ± 1.672 µg QE/mg dry extract, respectively. The commercial NP Ag flavonoid content was 0.01050 ± 0.01344 µg QE/mg dry extract (Table 1).

### 3.3. Characteristics of Synthesized NPs

**Figure 1:**
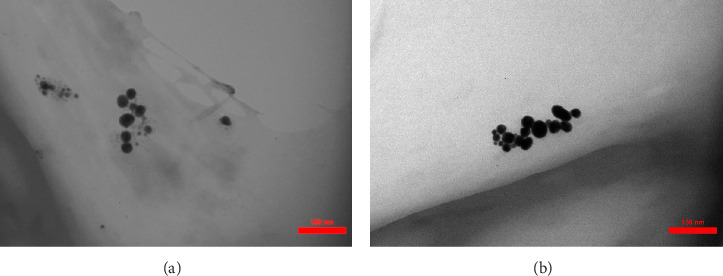
Transmission electron microscopy (TEM) images of (A) silver nanoparticles (Ag NPs) synthesized by hydroalcoholic *Echinophora platyloba* extract and (B) gold nanoparticles (Au NPs) synthesized by hydroalcoholic *E. platyloba* extract.

TEM imaging revealed that the average size of synthesized Au NPs (Au concentration of 0.5 mM) and Ag NPs (Ag concentration of 0.5 mM), using hydroalcoholic extracts of *E. platyloba*, was 31.42 ± 8.126 and 19.53 ± 0.914 nm, respectively. TEM also revealed that the form of the synthetic NPs was spherical (Figure 1).

**Figure 2:**
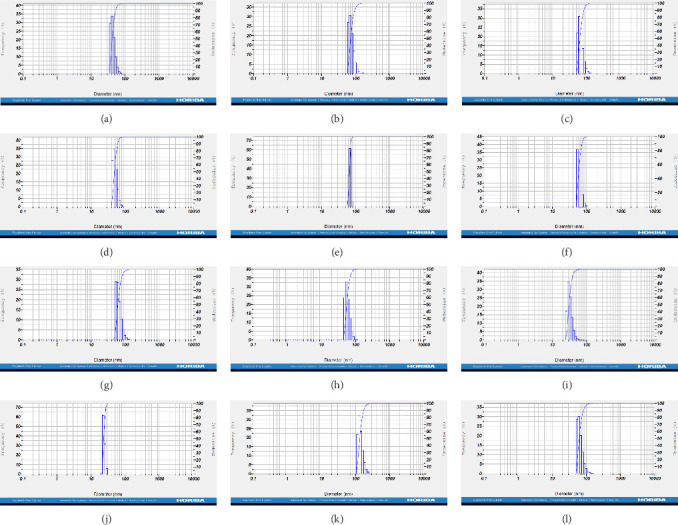
Dynamic light scattering (DLS) of, respectively, gold nanoparticles (Au NPs) hydroalcoholic extract Au concentration of 1 mM (A), Au NPs boiled extract Au concentration of 1 mM (B), Au NPs aqueous extract Au concentration of 1 mM (C), Au NPs hydroalcoholic extract Au concentration of 0.5 mM (D), Au NPs boiled extract Au concentration of 0.5 mM (E), Au NPs aqueous extract Au concentration of 0.5 mM (F), silver nanoparticles (Ag NPs) hydroalcoholic extract Ag concentration of 1 mM (G), Ag NPs boiled extract Ag concentration of 1 mM (H), Ag NPs aqueous extract Ag concentration of 1 mM (I), Ag NPs hydroalcoholic extract Ag concentration of 0.5 mM (J), Ag NPs boiled extract Ag concentration of 0.5 mM (K), and Ag NPs aqueous extract Ag concentration of 0.5 mM (L).

DLS measurements revealed that the mean size of synthesized Au NPs at an Au concentration of 1 mM, using hydroalcoholic, boiled, and aqueous extracts of *E. platyloba*, was 44.1 ± 7, 74.4 ± 14.5, and 66.7 ± 12.1 nm, respectively. For an Au concentration of 0.5 mM, the particle sizes with hydroalcoholic, boiled, and aqueous extracts were 49.9 ± 7.5, 66.4 ± 4.5, and 61.6 ± 8.5 nm, respectively. Moreover, the average size of Ag NPs synthesized at an Ag concentration of 1 mM, employing hydroalcoholic, boiled, and aqueous extracts, was 64.4 ± 12.9, 57.8 ± 9.6, and 32.1 ± 5.6 nm, respectively. For an Ag concentration of 0.5 mM, the particle sizes with hydroalcoholic, boiled, and aqueous extracts were 24.1 ± 1.9, 138.6 ± 25.8, and 65.7 ± 13.2 nm, respectively (Figure 2A–l).

The viscosity of the dispersion medium was measured at 0.894 mPa s and the zeta potential values for the 12 synthesized compounds were −38.4, −13.1, 12.3, −47.6, −19.2, −21.3, −40.2, −17.3, −23.1, −19.5, −15.7, and −16.3 mV, respectively.

**Figure 3:**
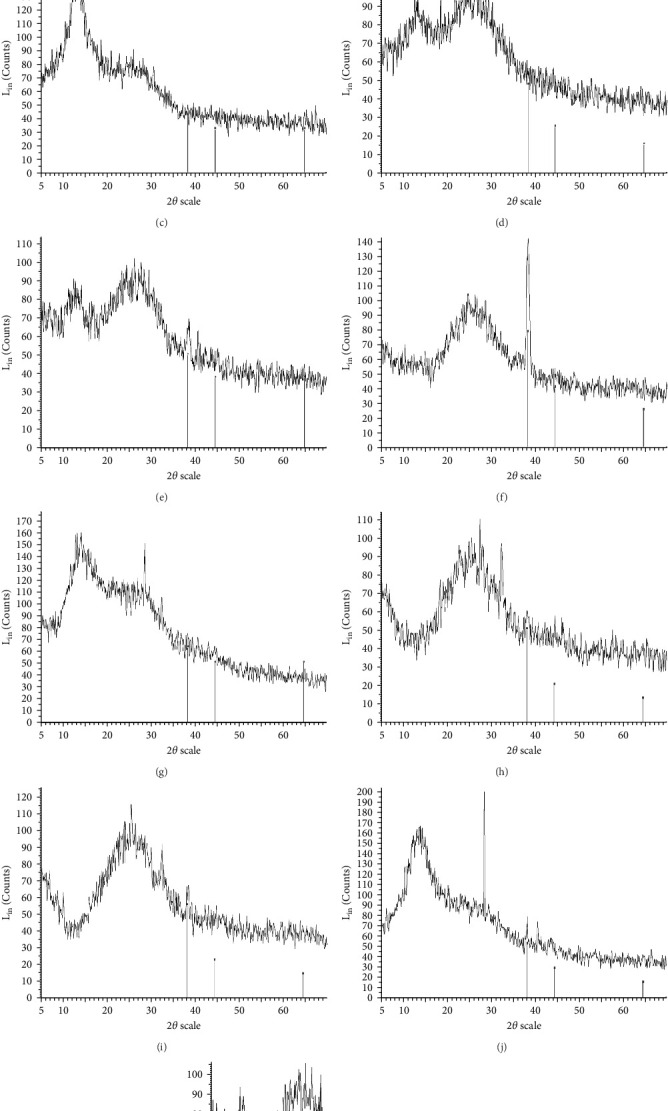
X-ray diffraction (XRD) of, respectively, gold nanoparticles (Au NPs) hydroalcoholic extract Au concentration of 1 mM (A), Au NPs boiled extract Au concentration of 1 mM (B), Au NPs aqueous extract Au concentration of 1 mM (C), Au NPs hydroalcoholic extract Au concentration of 0.5 mM (D), Au NPs boiled extract Au concentration of 0.5 mM (E), Au NPs aqueous extract Au concentration of 0.5 mM (F), silver nanoparticles (Ag NPs) hydroalcoholic extract Ag concentration of 1 mM (G), Ag NPs boiled extract Ag concentration of 1 mM (H), Ag NPs aqueous extract Ag concentration of 1 mM (I), Ag NPs hydroalcoholic extract Ag concentration of 0.5 mM (J), and Ag NPs aqueous extract Ag concentration of 0.5 mM (K).

The XRD patterns for all synthesized compounds are illustrated in Figure 3. Figure 3A–C correspond to the hydroalcoholic, boiled, and aqueous extracts of *E. platyloba* with a 1 mM Au solution. Additionally, Figure 3D–F illustrate the XRD patterns for the hydroalcoholic, boiled, and aqueous extracts of *E. platyloba* with a 0.5 mM Au solution. Figure 3G–I are associated with the hydroalcoholic, boiled, and aqueous extracts of *E. platyloba* with a 1 mM Ag solution, while Figure 3J,K pertain to the hydroalcoholic and aqueous extracts of *E. platyloba* with a 0.5 mM Ag solution.

The XRD pattern confirms the crystalline character of the synthesized NPs. The peaks show cubic crystalline characteristics of the NPs. The secondary peaks appeared due to the use of plant extracts, which, in contrast to chemical compounds, are not completely pure, so multiple peaks are observed in the XRD patterns.

FT-IR relies on alterations in the absorption and scattering patterns of near-infrared (NIR) radiation exhibited by the specimens. This analytical technique is employed to investigate the synthesis and characteristics of NPs.

**Figure 4:**
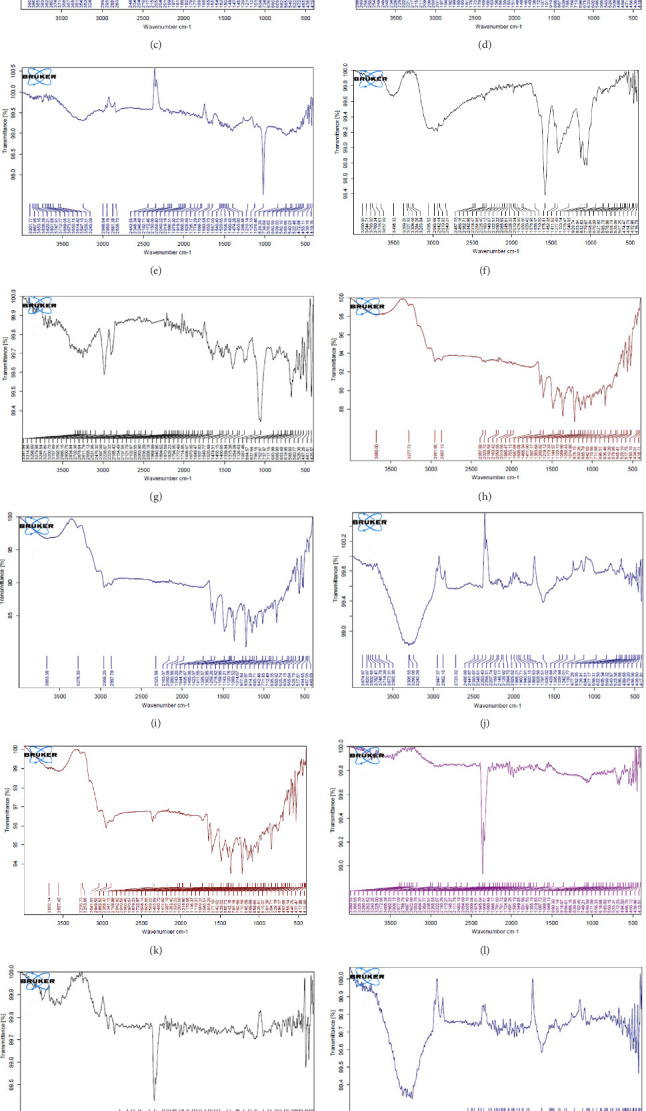
Fourier transform infrared (FT-IR) spectrum of hydroalcoholic *Echinophora platyloba* extract (A), gold nanoparticles (Au NPs) synthesized by hydroalcoholic extract with 1 mM Au (B), Au NPs synthesized by hydroalcoholic extract with 0.5 mM Au (C), silver nanoparticles (Ag NPs) synthesized by hydroalcoholic extract with 1 mM Ag (D), Ag NPs synthesis by hydroalcoholic extract with 0.5 mM Ag (E), boiled *E. platyloba* extract (F), Au NPs synthesized by boiled extract with 1 mM Au (G), Au NPs synthesized by boiled extract with 0.5 mM Au (H), Ag NPs synthesized by boiled extract with 1 mM Ag (I), Ag NPs synthesized by boiled extract with 0.5 mM Ag (J), aqueous *E. platyloba* extract (K), Au NPs synthesized by aqueous extract with 0.5 mM Au (L), Ag NPs synthesized by aqueous extract with 1 mM Ag (M), Au NPs synthesized by aqueous extract with 1 mM Au (N), and Ag NPs synthesized by aqueous extract with 0.5 mM Ag (O).

Figure 4A is the FT-IR spectrum of the hydroalcoholic extract of *E. platyloba*. As can be seen, there is a sharp absorption peak in the range of 1015 cm^−1^, which is related to cyclohexane groups as well as aliphatic fluoro compounds. The presence of a peak in the range of 1403 cm^−1^ is related to functional groups with O─H, such as phenols. The 2844 cm^−1^ range is connected to the O─CH functional group, and the 2944 cm^−1^ range is connected to the C─H functional group.

In Figure 4B,C, pertaining to the solutions of synthesized Au NPs, a comparative analysis with Figure 4. A reveals absorption percentages within the spectral range of 2360, 2343, and 1700 cm^−1^. These spectral bands are related to the C═C, ─O═C═O, and C═O functional groups, respectively. The mentioned spectral features suggest that particular functional groups have played a key role in the synthesis of Au NPs, effectively acting as capping agents on the Au surface.

In Figure 4D,E, existing dual absorption peaks at 2950 and 2881 cm^−1^ are due to the stretching vibrations (C─H) of secondary amine groups in the synthesized Ag NPs, as illustrated in Figure 4B,C. Additionally, absorption bands observed at 1650 cm^−1^ show the presence of amide groups in the NP solution. The absorption peak at 1398 cm^−1^ is a sign of C─H stretching in the plane of alkene and the bending of aldehyde groups. The FT-IR spectrum of the Ag NPs, biosynthesized with *E. platyloba* extract, reveals a notable absorption band at 1016 cm^−1^, assigned to polysaccharide sulfates (C─O─SO_4_), cyclohexane groups, and aliphatic fluoro compounds.

In addition, the absorption band at 834 cm^−1^ can be attributed to groups (RHC═CHR) in polymeric carbohydrates. The absorption band at 1650 cm^−1^ corresponds to the bending (N─H) of primary amine groups. Also, a strong vibrational mode at 1396 cm^−1^ noted in Ag NPs may be linked with the stretching mode (C─N) in the Ag nitrate bond. Moreover, the same peaks at 2881 and 2950 cm^−1^ in both the NP and Ag extract spectra correlate with stretching vibrations (C─H and C─C) related to alkane structures, amino, and alcoholic compounds. Small changes in peak positions compared to the plant extract show the involvement of primary constituents of *E. platyloba* extract, such as alcohols and phenolic ring compounds, in the reduction of Ag ions.

Figure 4F depicts the FT-IR spectrum of *E. platyloba* boiled extract. This specific illustration offers a comprehensive view of the distinctive peaks discerned in the *E. platyloba* extract, specifically at wavenumbers of 1046, 1123, 1411 1575, 2028, 2942, and 3498 cm^−1^. The C─N amine group could be the reason for the peak at 1046 cm^−1^. Also, the elongation of the ─C─O bonds present in phenolic acid or polysaccharides within the extract is the reason for the peak at 1123 cm^−1^. Additionally, the peak at 1411 cm^−1^ can be linked to the O─H alcohol group. The wavenumber of 1575 cm^−1^ is due to the stretching vibration of the C═C bonds, which could be a result of deformity in the aromatic ring of flavonoids. Moreover, alkynes and carbonyl groups, such as C═O, led to the peak at 2028 cm^−1^. The band at 2942 cm^−1^ seems to come from the stretching vibration of alkane groups, specifically CH_2_─ and CH_3_─ that are present in the extract. Finally, the band at 3498 cm^−1^ can be related to the aromatic rings and amide groups of polyphenols, triterpenoids, and flavonoids.

In Figure 4G,H, a comparative analysis with Figure 4F shows changes in the bands at 2986, 2951, 1400, 1363, 1219, 1051, and 842 cm^−1^ following the synthesis of Au NPs using *E. platyloba* boiled extract. This variation shows the effects of phytochemicals and functional groups in the extract and their part in the synthesis process of Au NPs.

Figure 4I,J exhibits noticeable shifts in spectral peaks at wavelengths of 3278, 2983, 1575, 1046, 977, and 712 cm^−1^, in contrast to the reference spectrum of the boiled extract (Figure 4F). This displacement functions as an indicative manifestation of the synthesis of Ag NPs utilizing the *E. platyloba* extract, thereby, suggesting that functional groups within the composition of the extract have played a substantial role in this synthesis process.

Figure 4K–O does not show any notable shifts in spectral peaks.

The UV-Vis spectrophotometer measurements of hydroalcoholic, boiled, and aqueous extracts of *E. platyloba*, in combination with an Au solution, were performed at three different points in time: 1, 2, and 24 h after the solutions were mixed. Having an absorption peak in the range of 520–530 nm reveals the formation of Au NPs in the extract. The UV-Vis spectrophotometer measurements of the hydroalcoholic, boiled, and aqueous extracts of *E. platyloba*, in combination with Ag NPs, were done at three different time points, about 1, 2, and 24 h after the solutions were mixed. The presence of an absorption peak in the range of 435–445 nm reveals the formation of Ag NPs in the extract.

### 3.4. In Vitro Antioxidant Effects

The *E. platyloba* extract showed the highest DPPH radical scavenging potential. The ability of green Ag NPs and green Au NPs to scavenge DPPH radicals was lower and even less for commercial Ag NPs (Table 2).

The *E. platyloba* extract has the highest antioxidant capacity; green Ag NPs showed a moderate antioxidant capacity. Green Au NPs had a lower FRAP value, and commercial Ag NPs had the lowest FRAP value (Table 2).

Green Ag NPs displayed the strongest RP. Conversely, commercial Ag NPs demonstrated the weakest RP. The *E. platyloba* extract and green Ag NPs showed intermediate RP values (Table 2).

### 3.5. In Vivo Antioxidant and Anti-Inflammatory Effects

Pretreatment with green Ag NPs (0.6 mg/kg) significantly (*p* < 0.01) lowered paw edema thickness, showing higher efficacy than indomethacin. Green Ag NPs and green Au NPs at a dose of 0.6 mg/kg were both effective to inhibit inflammation (*p* < 0.0001), showing similar activity to indomethacin. Green Ag NPs and green Au NPs at lower doses (0.3 mg/kg) were also effective to inhibit inflammation (*p* < 0.001). Administration of *E. platyloba* extract at a dose of 30 mg/kg suppressed inflammation (*p* < 0.01), while the *E. platyloba* extract at a dose of 15 mg/kg was less effective (*p* < 0.05; Table 3).

Carrageenan injection in the sub-plantar region of the paw significantly (*p* < 0.0001) increased the levels of the pro-inflammatory cytokines, IL-6 and TNF-*α* in mice paws. Pretreatment with *E. platyloba* extract, green Au NPs, green Ag NPs, and commercial Ag NPs significantly (*p* < 0.0001) lowered TNF-*α* cytokine levels. Administration of green Ag NPs at a dose of 0.6 mg/kg exhibited an effect similar to indomethacin treatment, with no significant differences observed compared to the control group. IL-6 levels were significantly lower in the groups treated with green Ag NP 0.3 mg/kg (*p* < 0.001), green Ag NP 0.6 mg/kg (*p* < 0.0001), green Au NPs 0.6 mg/kg (*p* < 0.0001), *E. platyloba* extract 15 mg/kg (*p* < 0.01), and *E. platyloba* extract 30 mg/kg (*p* < 0.05) compared to the carrageenan group (Table 4).

While carrageenan significantly decreased (*p* < 0.01) TAC levels, pretreatment with green Ag NPs 0.6 mg/kg significantly increased (*p* < 0.01) these levels compared to the carrageenan group. The MDA level in paw tissue was increased (*p* < 0.001) in the carrageenan group. Indomethacin effectively inhibited this increase (*p* < 0.01). The MDA levels were significantly lower (*p* < 0.05) in the groups receiving green Ag NPs 0.3 mg/kg, green Au NPs 0.6 mg/kg, and *E. platyloba* extract 30 mg/kg. Administration of green Ag NPs at a dose of 0.6 mg/kg had a more substantial effect in suppressing the increase (*p* < 0.001) in MDA level (Table 5).

## 4. Discussion

This experiment was developed to study the antioxidant and anti-inflammatory properties of green-synthesized Ag and Au NPs using *E. platyloba* extract.

DLS results showed that the NP synthesis was successful. DLS also revealed that the size of the synthesized NPs in all of the groups was less than 100 nm. The TEM images depict that the synthetic NPs are of spherical shape. The smallest and most homogeneous NP was synthesized using a hydroalcoholic extract, with Au and Ag at concentrations of 0.5 mM. The Ag NPs were even smaller and more uniform in comparison to Au NPs. The zeta analysis showed that the synthesized NPs have high negative or positive values, which indicate high stability and a low chance of aggregation [32]. The synthesized NP solutions have not shown signs of aggregation even after 6 months.

Our findings showed a high content of phenols and flavonoids in the *E. platyloba* extract and also in the synthesized NPs. FT-IR data also support the presence of these bioactive compounds and different functional groups, which could be responsible for reducing and stabilizing Ag and Au. These functional groups could be the reason for the antioxidant effect of the extract and synthesized NPs [33, 34]. FT-IR data also revealed that the NPs synthesized using the hydroalcoholic extract had more functional groups compared to the others.

After the synthesis process of NPs, we studied and compared the characteristic results of NPs from different extracts (boiled, aqueous, and hydroalcoholic) and different concentrations of Ag and Au (0.5 and 1 mM). This comparison led us to choose NPs synthesized using hydroalcoholic extract, with Au and Ag concentrations of 0.5 mM for the next studies.

In vitro antioxidant experiments, such as DPPH, FRAP, and RP and in vivo antioxidant evaluation methods like MDA and TAC correlate with other findings. Inflammation is a vital protective response that tissues utilize to respond to damaging inflammatory agents. The inflammatory mediators arrange all the inflammatory events, such as vasodilation, vascular permeability, leukocyte migration, and pain [35].

Carrageenan-induced paw edema has been accepted as a local and acute inflammation model. The inflammatory response in the mouse paw was the result of the injection of carrageenan and was characterized by increased paw volume and raised levels of pro-inflammatory cytokines, especially IL-6 and TNF-*α* [36]. Inflammation can cause oxidative stress and oxidative stress is able to activate pro-inflammatory pathways [37]. Therefore, the current experiment studied the anti-inflammatory and antioxidant effects of green Ag NPs, green Au NPs, and *E. platyloba* extract in an inflammation model.

The findings revealed that green Ag NPs, green Au NPs, and *E. platyloba* extract have significant efficacy in suppressing carrageenan-induced paw edema. Among these different treatments, green synthetic Ag NP 0.6 mg/kg showed the strongest antioxidant and anti-inflammatory effect. In our study, *E. platyloba* extract exhibited anti-inflammatory properties, which can be the result of the high amount of phenolic and flavonoid compounds present in this extract. Phenolic compounds, such as flavonoids, are known for their anti-inflammatory properties [38, 39]. The mechanism of these compounds is due to their ability to suppress the production or activation of pro-inflammatory mediators [40].

The current study revealed that green Ag NPs and green Au NPs significantly lowered the levels of IL-6 and TNF-*α* in mice paws. These findings support previous studies. In separate studies, Ag NPs synthesized using *Chamaemelum zeylanicum* extract and *Chamaemelum nobile* extract could reduce IL-6 and TNF-*α* levels in treatment groups [41, 42]. Another study showed that Au NPs synthesized by *Euphrasia officinalis* were able to lower IL-6 and TNF-*α* levels [43].

The precise anti-inflammatory mechanism of synthetic green NPs is yet to be fully explained, but it is proposed that the presence of bioactive molecules capping the NP surface might be the reason [44]. For instance, alkaloids can stop the expression of pro-inflammatory factors, such as histamines, cytokines, and lipid mediators [45]. Saponins suppress the expression of interleukin-1*β* (IL-1*β*), IL-6, TNF-*α*, and inducible nitric oxide synthase, which lead to their anti-inflammatory effect [46]. The anti-inflammatory properties of flavonoids are related to their ability to reduce the release of pro-inflammatory factors like IL-1*β*, IL-6, and TNF-*α* through the mitogen-activated protein kinase pathway [47].

The obtained results conform to previous studies about the anti-inflammatory properties of Ag NPs. For example, Ag NPs synthesized by *C. nobile* had an anti-inflammatory effect [42]. Synthesized Ag NPs using *Cinnamomum zeylanicum* were tested in polycystic ovarian syndrome rat models and showed anti-inflammatory effects [41]. Ag NPs synthesized by aqueous curcumin extract have been reported to be effective against arthritis disease inflammation [48]. Au NPs synthesized by *E. officinalis* were able to suppress the lipopolysaccharide-stimulated inflammation in RAW 264.7 macrophages [43]. Au NPs synthesized with *Suaeda japonica* leaf extract as a reducing agent also had anti-inflammatory properties [49].

Tissue destruction caused by oxidative stress can be evaluated by the end products of lipid peroxidation, like MDA, which is one of the numerous aldehydes produced through lipid peroxidation [50]. TAC reflects the collective ability of various antioxidants, including enzymes and nonenzymatic molecules, to neutralize reactive oxygen species (ROS). Low TAC levels usually indicate a reduced capacity to combat oxidative stress [51]. High ROS levels are a hallmark of chronic inflammation. Reducing oxidative stress with ROS scavengers has emerged as an efficient strategy for suppressing inflammation [52, 53].

The present study showed the antioxidative effects of green Ag NPs and green Au NPs. This was confirmed through in vitro examinations, such as DPPH, FRAP, and RP methods, which demonstrated the ability of these green NPs to scavenge free radicals. Additionally, measuring MDA and TAC levels using in vivo techniques provided more evidence supporting their antioxidative potential.

Previous reports also affirm that green Ag NPs and green Au NPs, in addition to their anti-inflammatory effects, possess antioxidant activity [54–56]. The results of our study demonstrated a dose-dependent effect, with synthetic Ag NPs at 0.6 mg/kg exhibiting the most pronounced anti-inflammatory and antioxidant properties.

The better performance of synthetic NPs, even though *E. platyloba* extract has a higher concentration of anti-inflammatory and antioxidant compounds, suggests a synergistic effect between Ag NPs and the phytochemicals present in the *E. platyloba* extract. Another study confirmed that Ag NPs synthesized by *Moringa oleifera* and *Azadirachta indica* were more powerful anti-inflammatory and antioxidant agents compared to the crude extracts [56]. FT-IR analysis confirmed the presence of various phytochemicals in the synthesized NPs. As mentioned earlier, phytochemicals are the potential reason for the higher anti-inflammatory and antioxidant activity of these NPs compared to commercial Ag NPs and *E. platyloba* extract. Additionally, the small size of the NPs helps them penetrate the small capillaries to reach the targeted tissues. This targeted delivery increases drug accumulation at the intended site, ultimately resulting in higher therapeutic efficacy [57].

Indomethacin is an effective nonsteroidal anti-inflammatory drug (NSAID). Though this drug has therapeutic benefits, indomethacin is linked to several serious adverse effects, including cardiovascular complications, gastrointestinal disturbances, and neurological impairments [58].

Compounds with antioxidant and anti-inflammatory properties are effective for optimal skin wound healing [59]. *Echinophora platyloba* extract, particularly the green-synthesized Ag and Au NPs, exhibits these beneficial effects. On the other hand, the surface-to-volume ratio of green NPs has increased, which can increase the availability of the drug at the site of action. Therefore, green synthetic NPs are considered good potential candidates for the local treatment of wounds. Additionally, these green NPs may be useful in managing the oxidative side effects of anticancer drugs due to their potent antioxidant and anti-inflammatory roles [60]. Of course, such clinical applications require further studies on toxicity and long-term stability.

## 5. Conclusion

In the present study, Ag and Au NPs were synthesized using *E. platyloba*. The resulting green-synthesized NPs were confirmed to be spherical by TEM. DLS data revealed that the mean size of the NPs was less than 100 nm, and zeta potential analysis demonstrated their stability. FT-IR analysis indicated the presence of bioactive compounds in the synthesized NPs. The presence of absorption peaks in specific ranges confirmed the formation of NPs in the UV-Vis study. The obtained XRD pattern illustrated the crystalline nature of the synthesized NPs. In vitro experiments, including DPPH, FRAP, and RP assays, revealed the antioxidant properties of both the extract and the NPs. For in vivo experiments, carrageenan-induced paw edema was employed, demonstrating dose-dependent antioxidant and anti-inflammatory effects of the extract and NPs. A comparison of all the data obtained in this study showed that both Ag and Au NPs exhibit stronger antioxidant and anti-inflammatory properties than the hydroalcoholic extract. Between Ag NPs and Au NPs, Ag NPs were found to be more potent antioxidant and anti-inflammatory agents, overall proving to be the better NPs. Economically, the synthesis of Ag NPs is significantly more affordable compared to Au NPs.

## Figures and Tables

**Table 1:** Quantification of TFC and TPC in *Echinophora platyloba* extract green synthetic Ag NPs, Au NPs, and commercial Ag NPs.

Sample	TPC (µg GAE/mg of dry extract)	TFC (µg QE/mg of dry extract)
Extract	25.84 ± 7.439^a^	51.64 ± 10.15^a^
Green Ag NPs	20.44 ± 1.404^a^	26.99 ± 0.5015^b^
Green Au NPs	18.42 ± 1.265^a^	25.8 ± 1.672^b^
Commercial Ag NPs	0.334 ± 0.5768^b^	0.0105 ± 0.01344^c^

*Note:* Values are expressed as mean ± standard error of the mean. The data were statistically analyzed using a one-way ANOVA and Tukey's post hoc test. Mean values followed by different superscript letters indicate a significant statistical difference (*p* < 0.05).

Abbreviations: Ag NPs, silver nanoparticles; Au NPs, gold nanoparticles; GAE, gallic acid equivalents; QE, quercetin equivalents; TFC, total flavonoid content; TPC, total phenol content.

**Table 2:** Antioxidant activity of *Echinophora platyloba* extract green synthetic Ag NPs, Au NPs, and commercial Ag NPs.

Sample	DPPH (IC50 µg/mL)	FRAP (FeSO_4_ E mg/mg DW)	RP (QE mg/mg DW)
Extract	178.5 ± 1.5^a^	0.07791 ± 0.08668^a^	0.0002108 ± 0.0001491^a,b^
Green Ag NPs	7597 ± 5678^a^	0.06963 ± 0.06248^a^	0.0003308 ± 0.0002286^a^
Green Au NPs	28380 ± 370.4^b^	0.03971 ± 0.04566^a^	0.0001135 ± 6.379e-005^a,b^
Commercial Ag NPs	106800 ± 5892^c^	0.000105 ± 0.0001418^a^	1.120e–006 ± 1.424e-007^b^

*Note:* Values are expressed as mean ± standard error of the mean. The data were statistically analyzed using a one-way ANOVA and Tukey's post hoc test. Mean values followed by different superscript letters indicate a significant statistical difference (*p* < 0.05).

Abbreviations: Ag NPs, silver nanoparticles; Au NPs, gold nanoparticles; DPPH, 2,2-diphenyl-1-picrylhydrazyl; DW, dry weight; FeSO_4_ E, FeSO_4_ equivalents; FRAP, ferric reducing/antioxidant power; QE, quercetin equivalents; RP, reducing power.

**Table 3:** Effects of Ag NPs, Au NPs, indomethacin, and *Echinophora platyloba* extract on an inflammation model.

Treatment regimen	Percent of inhibition (%)	Reduction of thickness (mm)
Carrageenan	0.004 ± 0.3122^d^	0.0040 ± 0.3122^b^
Indomethacin	31.44 ± 5.408^a,b^	1.0320 ± 0.2033^a^
Extract 15 mg/kg	16.13 ± 4.626^c^	0.4325 ± 0.1477^a,b^
Extract 30 mg/kg	17.13 ± 4.776^b,c^	0.7300 ± 0.2003^a,b^
Ag NPs 0.3 mg/kg	21.96 ± 6.906^a,c^	0.4750 ± 0.6397^a,b^
Ag NPs 0.6 mg/kg	32.18 ± 8.504^a^	0.8575 ± 0.3695^a^
Au NPs 0.3 mg/kg	21.21 ± 11.34^a,c^	0.4550 ± 0.2357^a,b^
Au NPs 0.6 mg/kg	24.85 ± 5.402^a,c^	0.5700 ± 0.4272^a,b^
Commercial Ag NPs	15.61 ± 3.784^c^	0.3900 ± 0.2754^a,b^

*Note:* Values are expressed as mean ± standard error of the mean. The data were statistically analyzed using a one-way ANOVA and Tukey's post hoc test. Mean values followed by different superscript letters indicate a significant statistical difference (*p* < 0.05).

Abbreviations: Ag NPs, silver nanoparticles; Au NPs, gold nanoparticles.

**Table 4:** Effects of Ag NPs, Au NPs, indomethacin, and *Echinophora platyloba* extract on IL-6 and TNF-*α* in an inflammation model.

Treatment regimen	IL-6 (pg/mg protein)	TNF-*α* (pg/mg tissue)
Control	7.327 ± 0.6479^f^	19.49 ± 10.05^e^
Carrageenan	21.22 ± 1.029^a^	134.7 ± 2.302^a^
Indomethacin	8.939 ± 0.5786^e,f^	18.62 ± 6.063^e^
Extract 15 mg/kg	15.25 ± 1.567^b,c^	48.83 ± 10.29^b,c^
Extract 30 mg/kg	15.43 ± 1.436^b,c^	44.6 ± 10.33^b,c^
Ag NPs 0.3 mg/kg	14.08 ± 0.5843^b,d^	48.66 ± 1.927^b,c^
Ag NPs 0.6 mg/kg	9.959 ± 1.252^d,f^	20.91 ± 8.628^d,e^
Au NPs 0.3 mg/kg	17.35 ± 2.555^a,b,c^	58.48 ± 10.5^b^
Au NPs 0.6 mg/kg	13.10 ± 0.578^c,d,e^	37.45 ± 2.291^c,d^
Commercial Ag NP	17.62 ± 2.489^a,b^	59.18 ± 6.516^b^

*Note:* Values are expressed as mean ± standard error of the mean. The data were statistically analyzed using a one-way ANOVA and Tukey's post hoc test. Mean values followed by different superscript letters indicate a significant statistical difference (*p* < 0.05).

Abbreviations: Ag NPs, silver nanoparticles; Au NPs, gold nanoparticles; IL-6, interleukin-6; TNF-*α*, tumor necrosis factor-*α*.

**Table 5:** Effects of Ag NPs, Au NPs, indomethacin, and *Echinophora platyloba* extract on MDA and TAC in an inflammation model.

Treatment regimen	MDA (mole/mL)	TAC (mole/mL)
Control	0.05796 ± 0.05823^b^	1.168 ± 0.4739^a^
Carrageenan	0.2005 ± 0.03968^a^	0.4599 ± 0.05956^b,c^
Indomethacin	0.08 ± 0.02798^b^	0.6835 ± 0.09931^a,c^
Extract 15 mg/kg	0.1289 ± 0.03723^a,b^	0.622 ± 0.1123^a,c^
Extract 30 mg/kg	0.1049 ± 0.05283^b^	0.8313 ± 0.1318^a,b^
Ag NPs 0.3 mg/kg	0.1055 ± 0.04279^b^	0.7996 ± 0.1784^a,c^
Ag NPs 0.6 mg/kg	0.07449 ± 0.03089^b^	1.19 ± 0.349^a^
Au NPs 0.3 mg/kg	0.1154 ± 0.04808^a,b^	0.5546 ± 0.2594^b,c^
Au NPs 0.6 mg/kg	0.1032 ± 0.04347^b^	0.9369 ± 0.2746^a,b^
Commercial Ag NPs	0.1248 ± 0.06846^a,b^	0.2375 ± 0.06775^c^

*Note:* Values are expressed as mean ± standard error of the mean. The data were statistically analyzed using a one-way ANOVA and Tukey's post hoc test. Mean values followed by different superscript letters indicate a significant statistical difference (*p* < 0.05).

Abbreviations: Ag NPs, silver nanoparticles; Au NPs, gold nanoparticles; MDA, malondialdehyde; TAC, total antioxidant capacity.

## Data Availability

The data that support the findings of this study are available on request from the corresponding author.
